# Alteration in ATR protein level does not account for the inherent radiosensitivity of HPV-positive head and neck squamous cell carcinoma

**DOI:** 10.1016/j.tranon.2025.102359

**Published:** 2025-03-14

**Authors:** Sibylla Kohl, Florentine S.B. Subtil, Vanessa Climenti, Houmam Anees, Ann C. Parplys, Rita Engenhart-Cabillic, Sebastian Adeberg, Ekkehard Dikomey, Ulrike Theiß

**Affiliations:** a Department of Radiotherapy and Radiation Oncology, Philipps-University Marburg, Marburg, Germany; b Marburg Ion-Beam Therapy Center (MIT), Department of Radiotherapy and Radiation Oncology, Marburg University Hospital, Marburg, Germany; c Laboratory of Radiobiology & Experimental Radiooncology, University Medical Center Hamburg Eppendorf, Hamburg, Germany

**Keywords:** Head and neck squamous cell carcinoma, Human papilloma virus, ATR, Radiosensitivity, DSB repair, HR

## Abstract

**Objectives:**

Human papilloma virus (HPV)-positive head and neck squamous cell carcinoma (HNSCC) cells are highly radiosensitive resulting from an elevated number of DNA double-strand breaks (DSB) remaining after irradiation. Partially this effect is due to a defective homologous recombination (HR). HPV-positive cells also show pronounced instability of chromosome 3, which codes for the kinase ataxia-telangiectasia and Rad3-related (ATR) protein, a central player of HR. If there is a contribution of ATR to the radiosensitivity of HPV-positive cells remains unclear, and this in-vitro study tested a functional involvement of ATR expression.

**Methods:**

The study was performed with six HPV-negative and six HPV-positive HNSCC cell lines. Gene copy number and gene expression were determined via qRT-PCR, protein expression by Western Blot. Response of cells towards irradiation in dependence of ATR expression was tested after siRNA Knock-down (ATRKD). Clonogenic survival after photon irradiation was evaluated by colony formation assay and DSBs were visualized by γH2AX/53BP1 co-staining.

**Results:**

ATR gene copy number and expression were not altered. Protein level was almost two-fold lower in HPV-positive compared to HPV-negative cells, but fully functional as observed by active phosphorylation in response towards irradiation. ATRKD resulted in a further increase in both, radiosensitivity as well as number of residual DSBs, but only for HPV-positive cells.

**Conclusion:**

Since the effect of ATRKD was compensated in HPV-negative but not in HPV-positive cells, these data revealed that the two-fold lower level of ATR in HPV-positive cells does not account for their enhanced inherent radiosensitivity, but acts additive to irradiation.

## Highlights

• HPV-positive HNSCC cells show lower ATR protein levels than HPV-negative.
• Despite low protein levels, ATR is fully functional and activated in response to radiation.
• HPV-positive but not HPV-negative cells are radiosensitized after ATR knockdown.
• HPV-positive cells are able to repair one-ended DSBs by homologous recombination.

## Introduction

Overall survival of HPV-positive (pos.) head and neck squamous cell carcinoma (HNSCC) reaches up to 80 % when treated by conventional radiochemotherapy [[1], [2], [3], [4]]. The good prognosis is considered to result from an enhanced cellular radiosensitivity of HPV-pos. compared to HPV-negative (neg.) cells, as evidenced by several publications which demonstrate a high number of DNA double-strand breaks (DSBs) as well as a pronounced G2-arrest remaining in HPV-pos. cells after irradiation [[5], [6], [7]]. These effects are suggested to arise from a defective homologous recombination (HR) [8,9] or Fanconi anaemia (FA) pathway [10]. HR is one of the two major DSB-repair pathways active in mammalian cells [11] and it was shown that the formation of Rad51 foci, which is a key step of HR, is strongly depressed in HPV-pos. cells. This was observed for both, cell cultures [8] as well as tumour biopsies [9].

Tumour cells are very well known for their elevated chromosomal instability (CIN) and also HNSCCs share these traits [12]. When measured by FISH, on average, level of CIN measured for HPV-neg. and -pos. HNSCC cells was identical [13]. There was, however, a clear difference in the chromosomes involved. For HPV-neg. cells translocations were formed almost randomly using all chromosomes, while for HPV-pos. cells chromosome 3 was significantly more involved than any other chromosome, demonstrating a particular instability of chromosome 3.

Among many other DNA repair-related genes, chromosome 3 also codes for the kinase ataxia-telangiectasia mutated and Rad3-related (ATR), which is the central protein for the repair of one-ended DSBs by HR [14,15]. This type of DSB is induced, when single-strand breaks collide with replication forks. It occurs in the context of DNA synthesis during S-phase of the cell cycle. Such a structure will lead to single-stranded DNA and cause an activation of ATR. ATR will then activate CHK1 to induce cell-cycle arrest as well as the phosphorylation of numerous other factors finally allowing the restart of the stalled replication forks.

Most DSBs, which are induced by genomic stress such as irradiation, are two-ended. This type of break can be repaired error-free by HR in late S- and G2-phase of the cell cycle, where an intact sister chromatid is present to perform this pathway [15,16]. However, in contrast to one-ended DSBs which require ATR, for two-ended DSBs HR is initiated by the recruitment of the kinase ataxia-telangiectasia mutated (ATM).

In this particular study, we were interested whether the elevated instability of chromosome 3 might lead to a defect in the expression or regulation of ATR thereby causing an impaired HR and concomitantly an enhanced radiosensitivity of HPV-pos. cells. For these experiments copy number, gene expression and protein level of ATR as well as its activation were measured for six HPV-pos. and six HPV-neg. cell lines, and also the effect of ATR knockdown (KD) on cell survival and DSB repair was examined by using specific siRNA transfection.

## Materials and methods

### Cell culture and consumables

The six HPV-neg. cell lines and the six HPV-pos. cell lines were provided by the following distributors: UM-SCC3, UM-SCC6, UM-SCC11b, UM-SCC104, UM-SCC47 (T. E. Carey, University of Michigan, United States); 93-VU147T (H. Joenje, VU Medical Center, Amsterdam, The Netherlands); UT-SCC33 (R. A. Grenman, Turku University, Finland); UD-SCC1, UD-SCC2 (H. Bier, University of Munich, Germany); UPCI:SCC152 (Susanne M. Gollin, University of Pittsburgh, United States); FaDu, UPCI:SCC154 (ATCC, Wesel, Germany). Cell line authentication via Short Tandem Repeats (STR) analysis was conducted at Helmholtz Center Munich using the GenePrint 10 kit (Promega, Mannheim, Germany) and GeneMapper 5.0 software. Comparison of the data with the Expasy and DSMZ databases verified the cell lines [17]. All cell lines were cultivated in RPMI-1640 medium (Sigma Aldrich, Munich, Germany) supplemented with 10 % FBS (Biochrom, Berlin, Germany), 2 mM l-glutamine (Capricorn Scientific GmbH, Ebsdorfergrund, Germany), 1 % non-essential amino acids (PAA Laboratories GmbH, Pasching, Austria) and 1 % penicillin/streptomycin (Capricorn Scientific GmbH, Ebsdorfergrund, Germany). OKF6 cell maintenance was done in a 2:1:1 mix of keratinocyte SFM:DMEM:F12 medium supplemented with 40 µg/mL BPE, 0.03 ng/mL hurEGF, 25 U/mL penicillin, 25 µg/mL streptomycin, and 155 µM CaCl_2_ (Life Technologies, Darmstadt, Germany). Culture conditions were 37 °C in a humidified 5 % CO_2_ atmosphere. Routine PCR testing for mycoplasma was negative [18].

### DNA copy number

DNA copy number was quantified by quantitative real-time PCR as described previously [19]. DNA was extracted using the NucleoSpin Tissue Kit (Macherey & Nagel, Düren, Germany). 40/8/1.6 ng of gDNA were added to a 20 µl SYBR Green mastermix containing 300 nM of primers. Relative quantification of the copy number was achieved by applying the standard curve method. Therefore, human placenta gDNA as standard normal tissue control was used (Promega, Mannheim, Germany). The qBiomarker copy number PCR assay multireference kit (Qiagen, Hilden, Germany) was utilized for normalization. Following primers were used (5´−3´): *ATR* forward AGCTTTGGAAAGAACCCGCT, *ATR* reverse AGAAGCCAGATTGCAAG-GGG.

### Gene expression

RNA was extracted using a NucleoSpin RNA II Kit (Macherey & Nagel, Düren, Germany) according to the manual. Reverse transcription per 500 ng total RNA was carried out with 200 U RevertAid Reverse Transcriptase in the presence of 5 mM random hexamers, 5 mM Oligo(dT)18, 500 mM dNTPs, and 1 U/mL RiboLock RNase inhibitor (all Thermo Scientific, Waltham, MA, USA). A QuantStudio5 Real-Time PCR System (Thermo Scientific) was used for detection. Relative quantification was analyzed by the ddCT method, normalizing to a housekeeper reference gene. Following primers were used (5´−3´): *ATR* forward GGTCACCACCAGACAGC CTAC, *ATR* reverse GAACATCACCCTTGGACCAGA, *18S* forward CGGCTACCACATCCAAG-GAA, *18S* reverse GCTGGAATTACCGCGGCT, *ALAS* forward TCCACTGCAGCA-GTACACTACCA, *ALAS* reverse ACGGAAGCTGTGTGCCATCT, *ß-actin* forward CCTGGCACCCAGCACAAT, *ß-actin* reverse GCCG ATCCACACGGAGTACTT.

### Protein expression

Protein detection was performed as described in [20]. Cells in exponential growth were prepared for western blot. Following antibodies were used for detection: ATR (1:1000, Santa Cruz Biotechnology Inc. #515173), pATR (1:1000, Cell Signaling #2853), ß-actin (1:5000, clone AC-15, Sigma-Aldrich #A5441), PIK3CA (1:1000, Invitrogen MA5–14870), BAP1 (1:500, Santa Cruz Biotechnology Inc. #28383), PARP3 (1:500, Santa Cruz Biotechnology Inc. #390771), MLH1 (1:1000, Invitrogen MA5–15431), TP63 (1:500, Santa Cruz Biotechnology Inc. #25268), VHL (1:500, Santa Cruz Biotechnology Inc. #135657), XPC (1:500, Santa Cruz Biotechnology Inc. #74410). Secondary antibodies were anti-rabbit/anti-mouse IgG HRP (horseradish peroxidase)-linked antibodies (1:5000, Millipore, Darmstadt, Germany). An ECL chemiluminescence detection system was used for visualization (Bio-Rad, Feldkirchen, Germany).

### siRNA transfection

Specific targeting of ATR by siRNA transfection was performed as described in [21]. Briefly, Lipofectamine 2000 transfection reagent (Life Technologies, Carlsbad, CA, USA) was used according to the manufacturer's instructions, with *ATR*-specific siRNA oligonucleotides (J-003202–19: GAGAAAGGAUUGUAGACUA, J-003202–20: GCAACUCGCCUAACAGAUA, J-003202–21: CCACGAAUGUUAACU-CUAU, J-003202–22: CCGCUAAUCUUCUAACAUU) or nontargeting siRNA (D-001810–01: UGGUUUACAUGUCGACUAA, D-001810–02: UGGUUUACAUGUUU-UCUGA, D-001810–03: UGGUUUACAUGUUGUGUGA, D-001810–04: UGGUUUA-CAUGUUUUCCUA) purchased from Dharmacon (ON-Targetplus, SMARTpool; Horizon Discovery Group, Cambridge, UK). 1–1.5 × 10^4^ cells/cm² were seeded, and after adherence followed transfection with an incubation time of 4–5 h in a mix of 20–50 nM siRNA and Lipofectamine in OPTI-MEM (Life Technologies, Carlsbad, CA, USA). Cells were validated for knockdown either 1 day after transfection or before irradiation.

### Irradiation

Cells were irradiated with X-rays using a Precision X-RAD 320ix (Precision X-ray, North Branford, CT, USA) at 320 kV and 8 mA. The dose rate was 1.1 Gy/min, and a Thoräus filter of 0.5 mm Cu + 0.5 mm Al was used. Absolute dose measurements confirmed the applied doses.

### Colony formation assay

For detection of clonogenic survival, cells were harvested in exponential growth and seeded as single cells in predefined cell numbers (4 × 10^2^ - 1 × 10^4^ per cm²). Cells were irradiated with indicated doses after 8 h adherence. Colonies were stained (10 % formaldehyde, 0.1 % crystal violet) after a growth period of 10–14 days. Only cell cluster >50 cells were considered for analysis. Plating efficiency was determined, and survival was calculated by normalizing to untreated cells.

### Immunofluorescence

Co-staining of γH2AX and 53BP1 was carried out to detect DSB repair foci as described previously [7]. Exponentially growing cells (1–2 × 10^4^ per cm^2^) were seeded on glass cover slips and left for adherence. Cells were treated with 0 or 2 Gy and residual foci were determined 24 h after irradiation. Fixation was performed in 4 % PFA/PBS for 10 min, followed by a permeabilization with 0.2 % Triton X-100, 1 % BSA/PBS for 10 min, washed with 1 % BSA/PBS and blocked in 3 % BSA/PBS for 1 h. Primary antibody solutions incubated for 1.5 h. Thereafter cells were washed three times 10 min with 0.1 % Tween20/PBS. Secondary antibody solutions incubated 1.5 h, followed by another three washing steps. Cover slips were mounted with ProLong Gold Antifade reagent with DAPI (Invitrogen, Karlsruhe, Germany). A Leica DM5500 wide-field microscope was used to analyse cell nuclei. Z-stacks were imaged using an immersion objective with 63x magnification and a numerical aperture of 1.25. Images were processed with the LAS-AF software (Leica, Wetzlar, Germany). Following primary antibodies were used: Anti-γH2AX (mouse, 1:500, Millipore, Darmstadt, Germany), Anti-53BP1 (rabbit, 1:500, Novus Biologicals, Wiesbaden, Germany). Following secondary antibodies were used: AlexaFluor488 donkey anti-rabbit IgG (*H* + *L*) and AlexaFluorPlus647 goat anti-mouse IgG (*H* + *L*) (both 1:1000, Invitrogen, Karlsruhe, Germany). The experiment was performed in two biological replicates and for each treatment condition at least 100 foci were counted as generally requested [https://radbiolab.shinyapps.io/terrific; accessed on 21 June 2022].

### Statistical analysis

The data is shown in mean values (MV) ± standard error of the mean (SEM). At least three independent biological experiments were performed, if not stated otherwise. Survival curves were fitted using the linear-quadratic model. The Student´s *t-*test was used for calculation of significance levels. Values for *p* < 0.05 were considered statistically significant. Data analysis was carried out using Excel and Graph Pad (GraphPad Software Inc., La Jolla, CA, USA) software.

## Results

### ATR characteristics in HPV- neg. and pos. cell lines

**Fig. 1:**
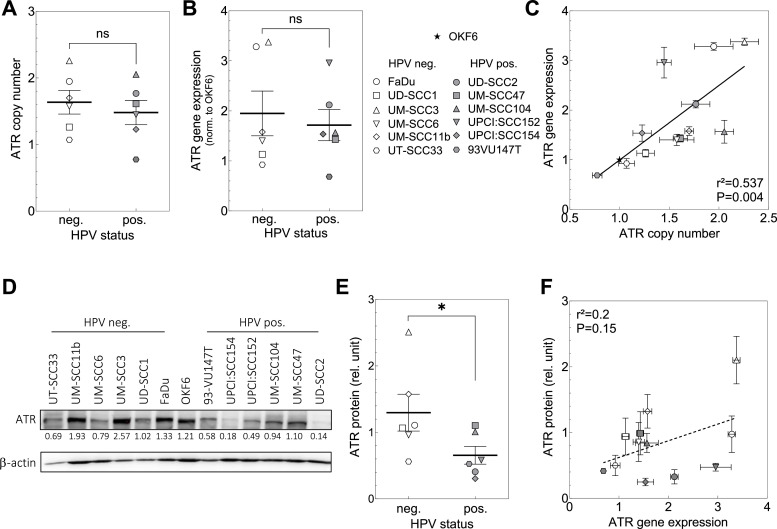
ATR in HPV neg. and pos. HNSCC cell lines. Cells in exponential growth were characterized on the genomic and protein level. (A) Gene copy number and (B) gene expression were determined by qRT-PCR. (C) Association between copy number and gene expression. (D) Western Blot and (E) relative expression levels of ATR protein. (F) Association between gene expression and ATR protein. For gene expression, values were normalized to the respective values determined for the reference cell line OKF6. For protein measurement densitometric quantification was performed, and the relative expression levels were corrected for background and loading. Data were analyzed by linear regression analysis. Data are presented as mean values ±SEM; *n* = 3; ✱, *p* < 0.05; ✱ ✱, *p* < 0.01, n.s.: non-significant.

For both, HPV-neg. and -pos. HNSCC cell lines the gene copy number of *ATR* as determined by qRT-PCR is increased, reflecting an aneuploidy as often seen in tumor cells (Fig. 1A). On average, there is a slightly but not significantly lower copy number in HPV-pos. cells (*P* = 0.555). A similar observation was made for the gene expression of *ATR* (Fig. 1B). Compared to expression in normal oral keratinocytes (OKF6), *ATR* expression is elevated in the HNSCC cells. Correlation between *ATR* copy number and gene expression (Fig. 1C) is highly significant. This finding demonstrates that the expression is mostly regulated via the copy number variation.

ATR protein expression exhibits inter-tumor cell variations (Fig. 1D) and a significant variation between the two entities (Fig. 1E). On average an almost twofold lower level of ATR protein was found for HPV-pos. cells (Fig. 1D; 1.28±0.20 vs. 0.70±0.14; *P* = 0.038). However, the level of protein did not correlate with the respective gene expression (Fig. 1F; r^2^=0.2, *p* = 0.15) indicating a dependence on other regulatory factors.

**Fig. 2:**
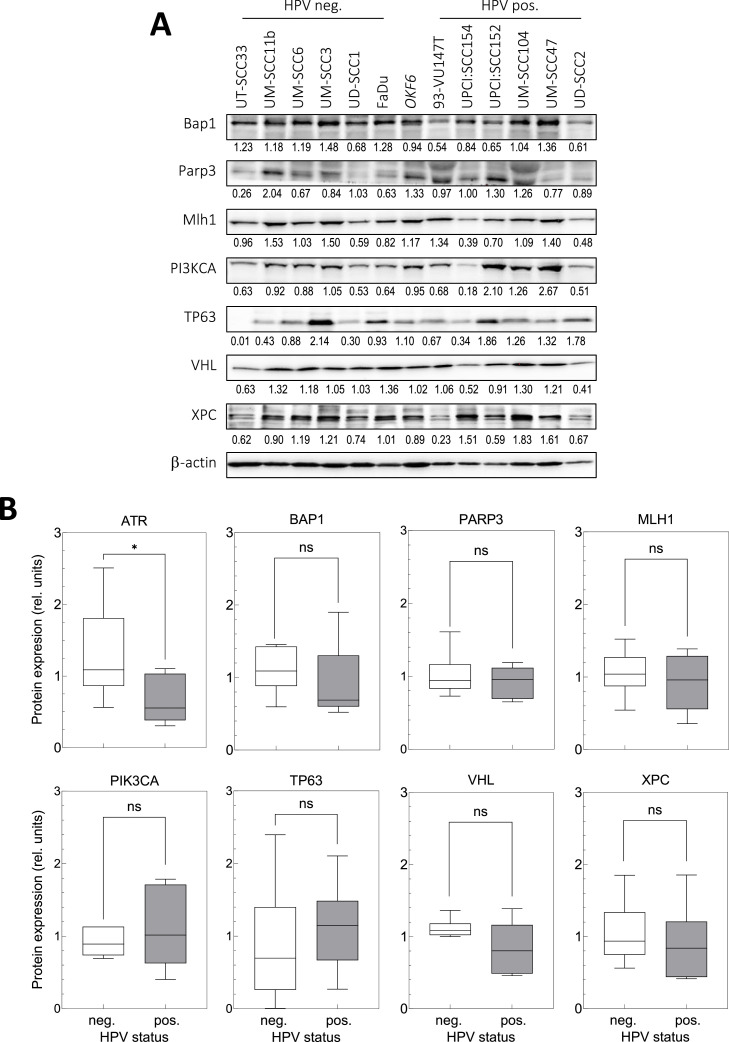
Variation of ATR protein in HPV neg. and pos. HNSCC cell lines in comparison to the protein of other genes located on chromosome 3. (A) Western blots, (B) Relative protein expression. Cells in exponential growth were prepared for western blot. Protein measurement was performed by densitometric quantification whereby relative expression levels were corrected to background and loading. Data are presented as box and whiskers showing the median ±quartile as well as min and max values; *n* = 3; ✱, *p* < 0.05; n.s.: non-significant.

In contrast to ATR, no significant difference in protein level was seen for seven other genes located on chromosome 3 (Fig. 2) and this was observed for both, genes located on the q-arm (*PI3KCA, TP63*) as well as the p-arm (*BAP1, PARP3, MLH1, VHL, XPC*) (Supplement Fig. 1). These data indicate that there is no general trend that the enhanced instability of chromosome 3 also affects the expression of respective genes.

### Activation of ATR

**Fig. 3:**
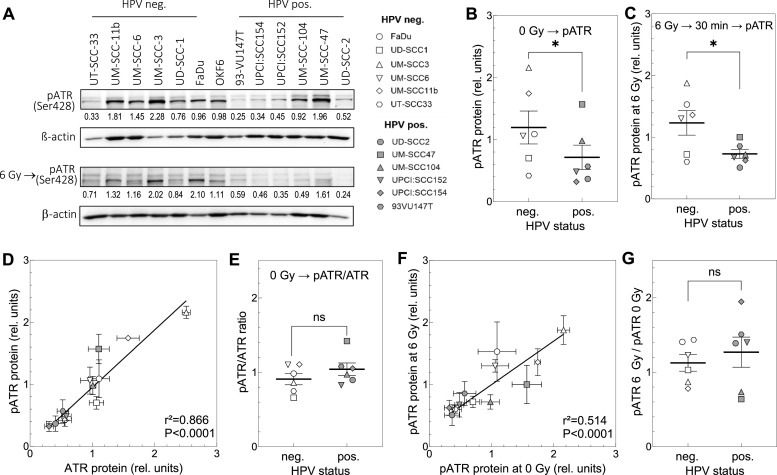
Activation of ATR in untreated and X-irradiated HPV-neg. and -pos. HNSCC cell lines. Cells in exponential growth were left untreated or irradiated with 6 Gy followed by an incubation for 30 min before cells were prepared for western blot. (A, B, C) Phosphorylation of ATR at serin 428. For protein measurement densitometric quantification was performed, and the relative expression levels were corrected to the background and loading. (D) Association of ATR protein and pATR for unirradiated cells. (E) Ratio of pATR / ATR. (F) Association between pATR measured for untreated cells and irradiated cells. (G) Respective ratio of pATR 6 Gy / pATR 0 Gy. (D, F) Data were analyzed by linear regression analysis. Data are presented as mean values ±SEM; *n* = 3; ✱, *p* < 0.05; ✱ ✱, *p* < 0.01, n.s.: non-significant.

The activation of ATR was measured by Western Blot for both, non-irradiated as well as irradiated cells using the phosphorylation site at Ser428 (Fig. 3A-C). In case of irradiation, pATR was determined 30 min after exposure to 6 Gy. For non-irradiated cells, ATR activation is more pronounced in HPV-neg. cells (Fig. 3B), and this difference can be observed after irradiation as well (Fig. 3C).

For non-irradiated cells, a straight correlation was seen between ATR and pATR for both HPV-neg. and pos. cell lines (Fig. 3D). These data indicate that the relative activation of ATR was the same for the two entities. In line with this, no significant difference was seen for the ratio of pATR/ATR between HPV-neg. and pos. cell lines (Fig. 3E).

There was also a straight correlation between pATR already detected in non-irradiated cells and the level of pATR reached in cells exposed to 6 Gy (Fig. 3F). The ratio pATR(6 Gy)/pATR(0 Gy) calculated from these data revealed that there was no difference between HPV-neg. and -pos. cell lines (Fig. 3G). Overall, these data demonstrate that albeit level of ATR is lower in HPV-pos. cells, the activation of ATR is not different from that measured for HPV-neg. cell lines neither for non-irradiated nor irradiated cells.

### Effect of ATR KD on radiosensitivity

In a next step we were interested in the effect of ATR inhibition on radiosensitivity. We aimed for a transient KD of *ATR* with siRNA (siATR) transfection, to guarantee for a highly specific downregulation. An efficient downregulation to about 10–20 % was achieved at a concentration of 20 nM si*ATR* and this effect lasted for at least 3 days (Supplement Fig. 2). Consequently, the experimental setup was designed in a way that ATR protein was not available during the period of DNA repair after irradiation.

**Fig. 4:**
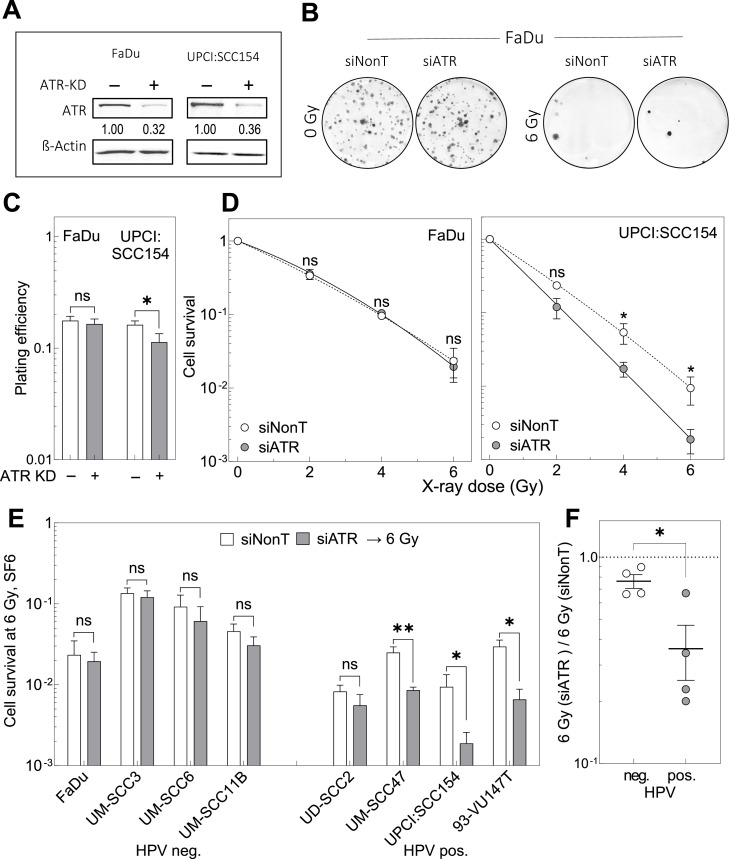
Effect of ATR KD on radiosensitivity of HPV-neg. and -pos. HNSCC cell lines. Cells in exponential growth were incubated with 20 nM siATR or siNonT, respectively. After 4 h medium was replaced followed by a further incubation for 20 h before cells were irradiated with X-ray doses up to 6 Gy and incubated for colony growth. (A) Western Blot for ATR KD validation. (B) Exemplary images of colony growth. (C) Effect of ATR KD on plating efficiency. (D) Cell survival of the HPV neg. cell line FaDu and HPV pos. cell line UPCISCC:154 after siATR or siNonT, followed by irradiation. (E) Cell survival of HPV neg. and pos. cell lines at 6 Gy with or without ATR KD. (F) Ratio of cell survival at 6 Gy SF6 (siATR) / SF6 (siNonT). KD of ATR see supplement Fig. 2. Data presented as mean values ±SEM; *n* = 3; ✱, *p* < 0.05; ✱ ✱, *p* < 0.01, n.s.: non-significant.

Effect of *ATR* KD on cell survival was determined by colony formation assay (Fig. 4A and B). *ATR* KD alone was found to have no effect on the plating efficiency of the HPV-neg. cell line FaDu, while a small yet not significant reduction was observed for the HPV-pos. cell line UPCI:SCC154 (Fig. 4C). When combined with radiation, no significant change was seen for FaDu in contrast to the significant increase in radiosensitivity for UPCI:SCC154 cells (Fig. 4D). An identical trend was observed for three other HPV-neg. and three HPV-pos. cell lines studied (Supplement Fig. 3). Using the data obtained at 6 Gy irradiation, a clear increase in radiosensitivity was measured for 3 out of 4 HPV-pos. cell lines in contrast to no significant change for all 4 HPV-neg. cell lines (Fig. 4E). Therefore, the ratio of survival at 6 Gy irradiation for SF6 (si*ATR*) / SF6 (si*NonT*) was significantly different for HPV-neg. and pos. cell lines (Fig. 4F, *P* = 0.016). These data suggest, that the effect of a downregulated ATR is compensated in HPV-neg. cells but not in HPV-pos. cells.

### Effect of ATR KD on DSB repair

**Fig. 5:**
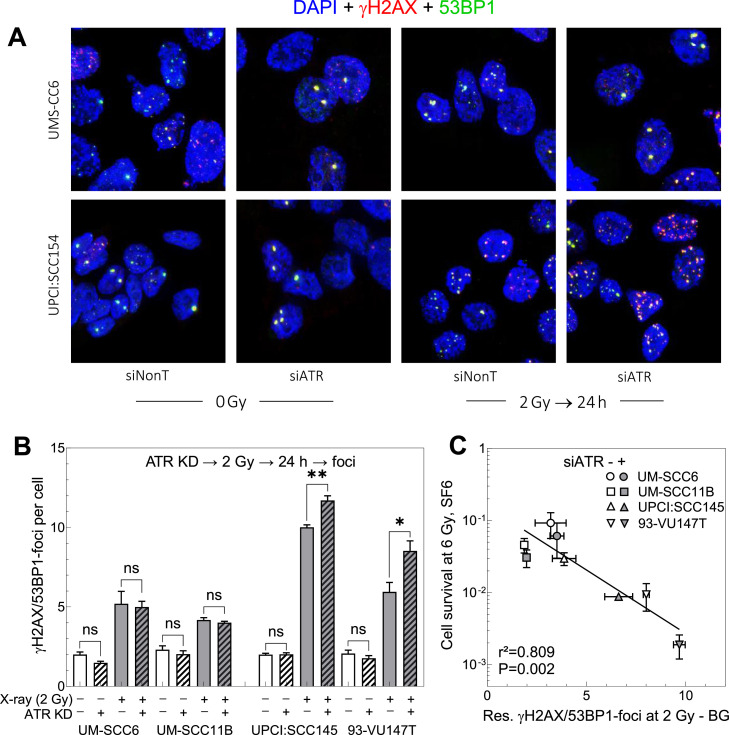
Effect of ATR KD on DSB repair of HPV-neg. and -pos. HNSCC cell lines. Cells in exponential growth were incubated with 20 nM siATR or siNonT, respectively. After 4 h medium was replaced followed by a further incubation for 20 h before cells were exposed either to 0 or 2 Gy X-rays followed by an incubation for 24 h before DSB-repair foci were detected by gH2AX/53BP1-foci assay. (A) Images of gH2AX/53BP1 foci in UM-SCC6 and UPCI:SCC154 cells. (B) Number of residual gH2AX/53BP1 foci 24 h after exposure to 0 or 2 Gy measured in cells treated w/wo ATR KD. (C) Association between residual gH2AX/53BP1 foci at 2 Gy subtracted by background and respective cell survival measured at 6 Gy. Data were analyzed by a log-linear regression analysis. Data presented as mean values ±SEM; *n* = 3; ✱, *p* < 0.05; ✱ ✱, *p* < 0.01, n.s.: non-significant.

Effect of *ATR* KD was also studied for DSB repair using co-staining of γH2AX and 53BP1 (Fig. 5A). Cells with or without *ATR* KD were irradiated with 2 Gy and the number of co-localised γH2AX/53BP1 foci were measured 24 h after irradiation. For non-irradiated cells *ATR* KD was found to have no significant effect on the number of foci, neither for the HPV-neg. cell lines UM-SCC6 and UM-SCC11b nor the two HPV-pos. cell lines UPCI:SCC154 and 93-VU147T (Fig. 5B). Number of foci strongly increased when cells were irradiated with 2 Gy, with approximately 2 times more residual foci in HPV-pos. over HPV-neg. cells (UM-SCC6: 5.2 and UM-SCC11B: 4.2 versus UPCI:SCC154: 10 and 93-VU147T: 6 foci). *ATR* KD prior to irradiation resulted in a further increase of residual foci, but this effect was solely observed for HPV-pos. cells (Fig. 5B; UPCI:SCC154: 11.7 and 93-VU147T: 8.5 foci). These changes reflect the different effects for HPV-neg. and pos. cells in respect to cell killing (Fig. 4). In line with this, a straight correlation was found between the increase in residual DSB-repair foci and the respective increase in cell killing (Fig. 5C). These data demonstrate, that the increase in radiosensitivity seen for HPV-pos. cells after *ATR* KD, resulted from an elevated number of residual DSB repair foci.

## Discussion

It was the aim of this project to test, whether the enhanced instability of chromosome 3 previously found for HPV-pos. cells [13], may lead to dysregulated or reduced expression of ATR thereby causing the enhanced radiosensitivity of these cells. The study was performed with six HPV-pos. and six HPV-neg. HNSCC cell lines to cover the great heterogeneity generally seen for these two entities [13,22,23].

For HPV-neg. and HPV-pos. HNSCC cells, there was neither a difference for the copy number nor the gene expression of ATR. There was, however, a significantly lower level of ATR protein in HPV-pos. cells. Such a difference was previously already observed by Moeller et al. [24] by analysing biopsies of 53 HPV-neg. and 36 HPV-pos. tumour samples. Also, the data presented by Kocher et al. [25] obtained for 5 HPV-neg. and 6 HPV-pos. cell lines indicated lower ATR levels for HPV-pos. cells. ATR protein was examined in the context of Cisplatin treatment by Leonard et al. [22] for 17 HPV-neg. and 4 HPV-pos. cell lines, but no test was made for the difference in ATR protein expression between the two entities. However, like the data shown here, a very low level of ATR was seen for the HPV-pos. cell line UD-SCC2 and an intermediate value for UM-SCC47 and 93-VU147T when compared to the HPV-neg. cell lines studied.

In contrast to ATR, no difference in protein level was observed for seven other genes located on chromosome 3 (Fig. 2). This finding indicates that the instability previously shown for chromosome 3 [13] does not lead to a general defect in gene expression.

Although ATR protein amount was less in HPV-pos. cells, downregulation by siRNA was still found to enhance the clonogenic cell death as well as the number of residual DSBs, indicating a clear radiosensitizing effect. These findings argue for a synergistic effect of the HR deficiency and the ATR KD in HPV-pos. cells. Downregulation of ATR is considered to specifically depress the repair of replication-mediated one-ended DSBs, which in turn can cause replication stress and additional DSBs [26,27]. Most likely, HPV-neg. cells are able to compensate ATR deficiency by other DSB repair pathways. Similar observations were made when replication stress was induced in HR-competent cells by Olaparib [28]. HR-deficient HPV-pos. cells apparently are not able to compensate for ATR deficiency. As a result, further reduction of ATR synergizes with the immanent repair defect and enhances the radiosensitivity. The reduced ATR protein level is not causative of the inherent radiosensitivity of HPV-pos. cells, it can rather be used to further enhance the treatment effect.

It is a fact that HPV-pos. cells are defective in HR as indicated by a depressed Rad51 foci formation post irradiation [8,9]. So far, it is not known whether this is due to a defect in the repair of one-ended DSBs by HR as mediated by ATR or the repair of two-ended DSBs by HR as mediated by ATM. The observation made here for HPV-pos. cells, that KD of ATR led to a further increase in radiosensitivity suggests that the repair of one-ended DSBs by HR appears to be intact in HPV-pos. cells. This argues for a defective ATM-dependent HR in HPV-pos. cells, and a recent publication supports this idea. The authors could show a defect in ATM-mediated DNA damage response which is independent from ATM expression level [25]. Consequently, they also would not find a correlation of ATM expression level and patient survival [29]. To understand the functions on a molecular level, further exploration is necessary.

To our knowledge, no other data is available about the effect of ATR KD on radiosensitivity in HPV-pos. cells using siRNA. There are, however, several other studies using various ATR inhibitors such as AZD6738. This inhibitor is known as a potent radiosensitizer and is already tested in several clinical studies [[30], [31], [32], [33]]. For HNSCC cells generally a moderate increase in sensitivity towards DNA damaging agents such as radiation or cisplatin was seen when adding ATR inhibitors. The magnitude of increase, however, appears to be subjected to larger variation for HPV-neg. cells when compared to HPV-pos. cells [22,[34], [35], [36]].

ATR inhibitors are considered to inhibit primarily by blocking the checkpoint regulation [27,37,38], only for AZD6738 a dysfunction in HR could be detected [34]. However, specificity is variable and dose-dependent, and some inhibitors can target PI3K, AKT or ATM, which will also affect radiosensitivity [31]. In a recent report [39], which used AZD6738 for ATR inhibition in HPV-negative cell lines, a far less radiosensitizing potenial of the ATR inhibitor was observed compared to inhibitors of ATM or DNA-PKcs. To avoid any probable side effects, we used siRNA in this study for KD of ATR. In this experimental setup, the moderate increase in radiosensitivity seen for HPV-neg. cells when using ATR inhibitors, was not observed when ATR is downregulated by specific siRNA.

## Conclusion

The enhanced inherent radiosensitivity observed for HPV-pos. cells is attributed to the elevated number of residual DSBs remaining after irradiation, which is suggested to result from a defect in the repair of DSBs by HR. It was now shown for six HPV-pos. HNSCC cell lines that on average level of ATR, which is a central player of HR, is only half of that measured in HPV-neg. cell lines. There is, however, no defect in ATR activation neither in untreated nor irradiated cells. KD of ATR by siRNA was found to result in a further increase of both radiosensitivity as well as number of residual DSBs but only for HPV-pos. and not for HPV-neg. cells. These findings demonstrate that the reduced level of ATR found for HPV-pos. cells does not account for their immanent radiosensitivity, in fact it fuels additional cell death. The fact that ATR KD cannot be compensated in HPV-pos. cells also revealed that these cells appear to be not defective in the repair of one-ended DSBs by HR, which is mediated by ATR. The immanent deficit is more likely in the repair of two-ended DSBs by HR, which depends on ATM.

## Financial support

This work was supported by the Anneliese-Pohl promotion of State doctorate of the Philipps-University Marburg,

## CRediT authorship contribution statement

**Sibylla Kohl:** Writing – review & editing, Validation, Investigation. **Florentine S.B. Subtil:** Writing – review & editing, Validation, Supervision. **Vanessa Climenti:** Investigation. **Houmam Anees:** Investigation. **Ann C. Parplys:** Investigation. **Rita Engenhart-Cabillic:** Writing – review & editing, Resources, Funding acquisition. **Sebastian Adeberg:** Writing – review & editing, Resources. **Ekkehard Dikomey:** Writing – original draft, Visualization, Validation, Supervision, Conceptualization. **Ulrike Theiß:** Writing – original draft, Visualization, Validation, Supervision, Methodology, Funding acquisition, Conceptualization.

## Declaration of competing interest

The authors declare that they have no known competing financial interests or personal relationships that could have appeared to influence the work reported in this paper.

## Appendix Supplementary materials

Image, application 1

Image, application 2
