# Supplementary material for: Alteration in ATR protein level does not account for the inherent radiosensitivity of HPV-positive head and neck squamous cell carcinoma
Source: Transl Oncol. 2025 Mar 14;55:102359. doi: 10.1016/j.tranon.2025.102359 (PMC11957528; doi:10.1016/j.tranon.2025.102359)
Supplement: Supplementary file 1 [file mmc1.pdf]

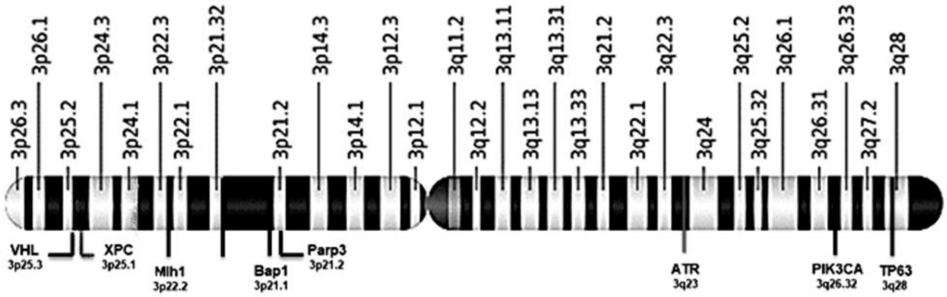

Supplementary Figure 1. Location of the different genes located on chromosome 3.

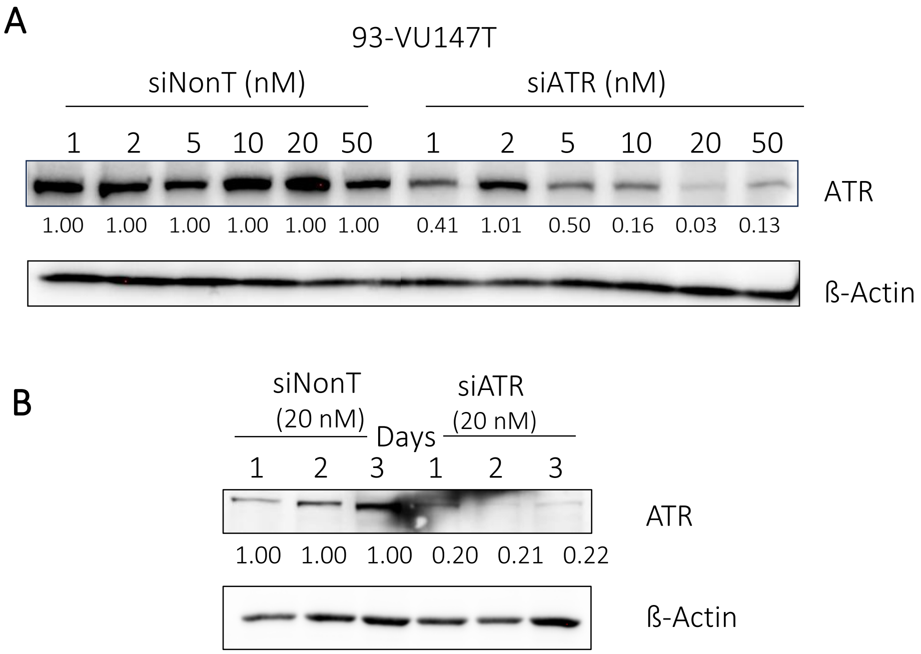

Supplementary Figure 2. Knockdown of ATR by siRNA. 93-VU147T cells in exponential growth were incubated with specific siRNA (siATR) as well as non-targeted siRNA (siNonT). After 4 h medium was replaced followed by a further incubation for 20 h before cells were prepared for western blot. Protein measurement was performed by densitometric quantification whereby relative expression levels were corrected for background and loading. (A) Treatment at concentrations ranging from 0 to 50 nM. (B) Treatment with 20 nM siNonT or siATR for 4 h was followed by an incubation ranging from 1 to 3 days. Protein measurement was performed by densitometric quantification whereby relative expression levels were corrected for background and loading and normalized to the control.
