# Supplementary material for: Alteration in ATR protein level does not account for the inherent radiosensitivity of HPV-positive head and neck squamous cell carcinoma
Source: Transl Oncol. 2025 Mar 14;55:102359. doi: 10.1016/j.tranon.2025.102359 (PMC11957528; doi:10.1016/j.tranon.2025.102359)
Supplement: Supplementary file 2 [file mmc2.pdf]

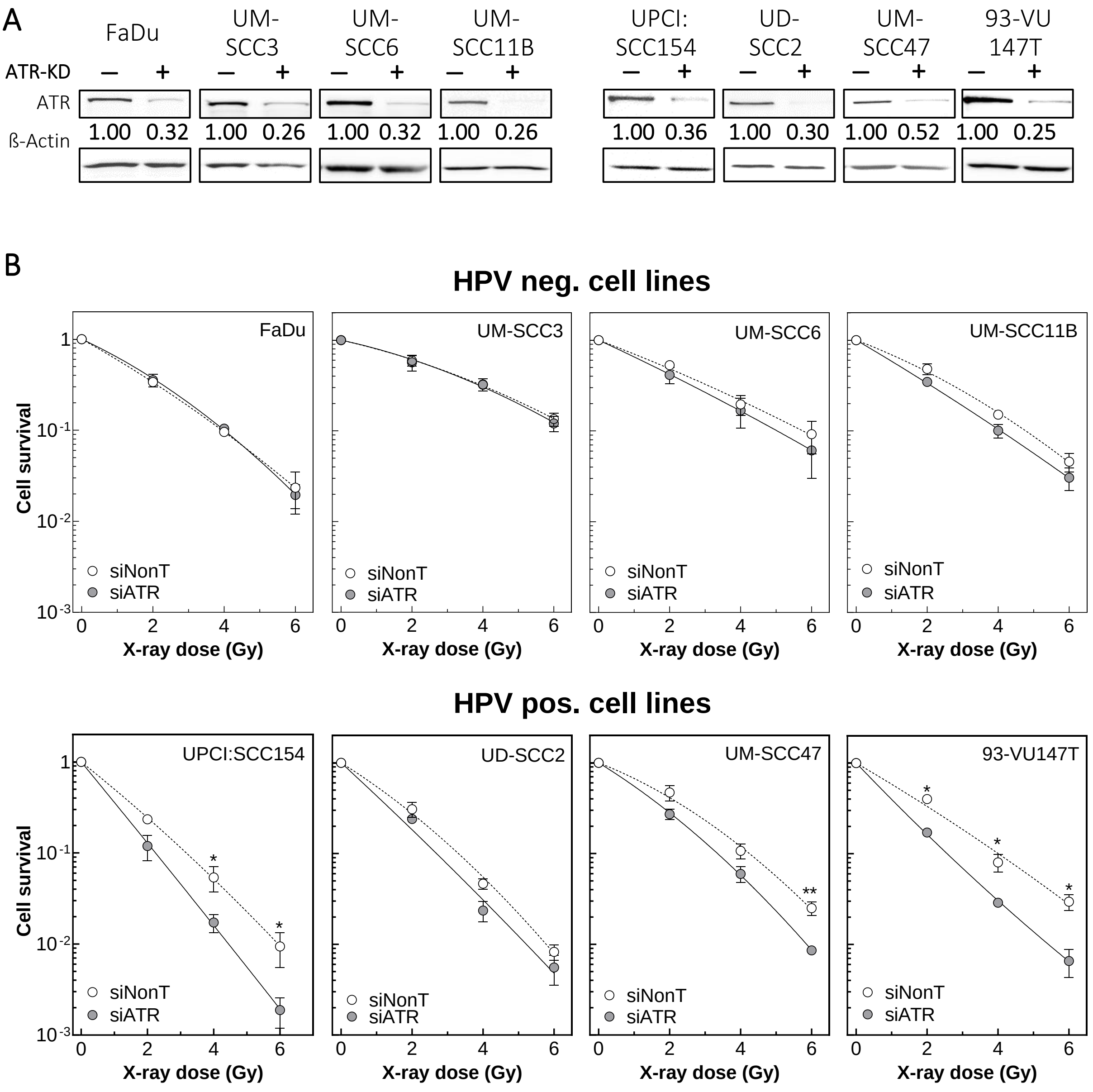

Supplementary Figure 3. Effect of ATR KD on radiosensitivity of HPV neg. and pos. HNSCC cell lines. Cells in exponential growth were incubated with 20 nM siRNA or siNonT, respectively. After 4 h medium was replaced followed by a further incubation for 20 h before cells were irradiated with X-ray doses up to 6 Gy and incubated for colony growth. (A) KD of ATR. (B) Cell survival after irradiation of the HPV neg. and pos. cell lines with or without ATR KD. Data presented as mean values ± SEM; n = 3; ✱, $p < 0.05$; ✱ ✱, $p < 0.01$, n.s.: non-significant.
